# Selective Modulation of Endoplasmic Reticulum Stress Markers in Prostate Cancer Cells by a Standardized Mangosteen Fruit Extract

**DOI:** 10.1371/journal.pone.0081572

**Published:** 2013-12-18

**Authors:** Gongbo Li, Sakina M. Petiwala, Dana R. Pierce, Larisa Nonn, Jeremy J. Johnson

**Affiliations:** 1 Department of Pharmacy Practice, College of Pharmacy, University of Illinois at Chicago, Chicago, Illinois, United States of America; 2 Department of Pathology, College of Medicine, University of Illinois at Chicago, Chicago, Illinois, United States of America; 3 University of Illinois Cancer Center, College of Medicine, University of Illinois at Chicago, Chicago, Illinois, United States of America; University of Nebraska Medical Center, United States of America

## Abstract

The increased proliferation of cancer cells is directly dependent on the increased activity of the endoplasmic reticulum (ER) machinery which is responsible for protein folding, assembly, and transport. In fact, it is so critical that perturbations in the endoplasmic reticulum can lead to apoptosis. This carefully regulated organelle represents a unique target of cancer cells while sparing healthy cells. In this study, a standardized mangosteen fruit extract (MFE) was evaluated for modulating ER stress proteins in prostate cancer. Two human prostate cancer cell lines, 22Rv1 and LNCaP, and prostate epithelial cells (PrECs) procured from two patients undergoing radical prostatectomy were treated with MFE. Flow cytometry, MTT, BrdU and Western blot were used to evaluate cell apoptosis, viability, proliferation and ER stress. Next, we evaluated MFE for microsomal stability and anti-cancer activity in nude mice. MFE induced apoptosis, decreased viability and proliferation in prostate cancer cells. MFE increased the expression of ER stress proteins. Interestingly, MFE selectively promotes ER stress in prostate cancer cells while sparing PrECs. MFE suppressed tumor growth in a xenograft tumor model without obvious toxicity. Mangosteen fruit extract selectively promotes endoplasmic reticulum stress in cancer cells while sparing non-tumorigenic prostate epithelial cells. Furthermore, in an in vivo setting mangosteen fruit extract significantly reduces xenograft tumor formation.

## Introduction

The increased proliferation of cancer cells is directly dependent on the increased activity of the endoplasmic reticulum (ER) machinery which is responsible for protein folding, assembly, and transport [1], [2]. In fact, it is so critical that modulation of protein synthesis if not regulated properly can lead to apoptosis [3]–[6]. This is especially true for cancer cells which have accumulated an ability to overcome cell cycle and apoptotic checkpoints [7]. As the demand for protein synthesis increases there is a proportional increase in “translational sloppiness” leading to the accumulation of unfolded and mis-folded proteins altering ER homeostasis. If left unchecked, these cells would undergo apoptosis, however, it is clear they have no intent in doing so. As expected, cancer cells develop a workaround utilizing a signal transduction pathway known as the “unfolded protein response (UPR)”. This process can alter the transcription and translation of proteins thereby re-establishing ER homeostasis - to a degree. This in turn promotes resistance to apoptosis and increases cell survival. This phenomenon is well established across many different cancers including cancer of the prostate.

The predominant theory of the UPR, which is regulated by several different ER stress proteins/pathways, is that a positive modulation of ER stress will promote survival [2]. In essence, this is true, however, what may be more significant is the degree to which ER stress proteins are modulated. As evidenced by our studies included herein, as well as studies by other investigators, we present data suggesting that a significant increase in ER stress proteins will result in apoptosis in cancer cells.

For our studies we evaluated an extract from the mangosteen (*Garcinia mangostana*) fruit and found it to significantly modulate ER stress proteins. Even more interesting was the seemingly specific response to increase ER stress proteins in prostate cancer cells while decreasing ER stress proteins in prostate epithelial cells. This effect represents a complex relationship that exists in non-cancerous and cancerous cells. Here we present evidence that an increase in the ER stress response protein CHOP/GADD153 is a viable approach to new strategies for therapeutics development.

## Materials and Methods

### Mangosteen fruit extract, kits and antibodies

Mangosteen Fruit Extract (MFE) was obtained from Avesthagen, Inc. (Chatsworth, CA). All antibodies for Western blot analysis, BrdU cell proliferation assay kit, and cleaved caspase-3 ELISA kit were obtained from Cell Signaling Technology (Danvers, MA). BCA Protein assay kit and chemiluminescent substrates were obtained from Pierce (Rockford, IL). APO-DIRECT™ kit was obtained from Phoenix Flow Systems (San Diego, CA). One-Step RT-PCR kit was obtained from Life Technologies (Grand Island, NY). RNeasy mini kit and RNase-Free DNase set were obtained from QIAGEN (Santa Clarita, CA).

### Liquid chromatography

For determining the concentration of α-mangostin in the mangosteen fruit extract samples were analyzed on a HPLC waters 2695 separation module. Separations were achieved using a gradient as previously described [8]. For determining the serum levels of α-Mangostin, serum samples were analyzed on a Thermoseparation SpectraSystem P4000 pump with an AS 3000 autosampler, a Thermoseparation SpectraSystem UV2000 detector at 243 nm, running time 20 min, and 10 µl of injection volume at room temperature. Separations were achieved with an isocratic solvent system, at 1.0 ml/min, and a Capcell Pak C-18, 3 µm, 4.6×250 mm column with inline Upchurch pre-column filter. The limit of quantitation and detection is 0.039 µg/ml (i.e. 95 nM), and 0.0098 µg/ml (i.e. 23.9 nM), respectively.

### Cell culture and treatment

LNCaP and 22Rv1 cells were obtained from American Type Culture Collection (Manassas, VA). These cells were cultured in RPMI supplemented with 10% fetal bovine serum (FBS) and 1% penicillin/streptomycin. All cells were maintained under standard cell culture conditions as described previously [9]. Cells were cultured in above-mentioned medium supplemented with a range of doses of MFE for desired times, then ready for downstream experiments.

### Primary prostatic epithelial cells and treatment

Primary prostatic epithelial cells (PrECs) were established from radical prostatectomy tissue at the University of Illinois at Chicago Medical Center as described previously [10]. Fresh tissue from the peripheral zone was selected by a pathologist according to an IRB-approved protocol. Briefly, the tissue was digested in collagenase, and plated on collagen-coated dishes in PrEGM media (Lonza, Walkersville, MD) for epithelial cell outgrowth. All cells were used at secondary passage and ∼70% confluency (cell density). PrECs were treated with 15 µg/ml MFE for 24 hr followed by downstream experiments.

### Cell viability

Cell viability was determined by 3-(4, 5-dimethylthiazol-2-yl)-2, 5-diphenyltetrazolium bromide (MTT) assay as described previously [11].

### Cell proliferation

Cell proliferation was determined by BrdU assay according to the manufacturer's manual.

### Flow cytometry

The APO-DIRECT™ kit (Phoenix Flow Systems, San Diego CA) was used for measuring apoptosis of treated cells by flow cytometry [11]. Briefly, 22Rv1 cells were plated to 50–60% confluence and then treated with complete media containing MFE. The kit was followed per protocol directions.

### Western blots

Whole cell lysates from treated cells were prepared as previously described [9], [12]. Lysates were quantified using BCA assay according to manufacturer's manual. A common western blots protocol was followed. Briefly, same amount of lysates were loaded to each well of 12% pre-cast gels (Bio-Rad, Hercules, CA). After transfer, membranes were blocked and incubated with primary antibody (1∶1000) overnight at 4°C, rinsed briefly, then incubated with secondary antibody (1∶2000) at room temperature for 1 hr. Membranes were washed, incubated with substrates and exposed in a FluorChem E imager (ProteinSimple, Santa Clara, CA).

### RT-PCR

Primers for XBP-1 and GAPDH RT-PCR were synthesized by IDT (Coralville, IA) according to previous studies [13]. Sequences were; XBP-1 forward, 5′-TTA CGA GAG AAA ACT CAT GGC C-3′, XBP-1 reverse, 5′-GGG TCC AAG TTG TCC AGA ATG C-3′, GAPDH forward, 5′-ACC ACA GTC CAT GCC ATC AC-3′, GAPDH reverse, 5′-TCC ACC ACC CTG TTG CTG TA-3′. 22Rv1 and LNCaP cells were treated with 15 µg/ml MFE for 24 hr. Total RNA was extracted from treated and control cells. One microgram of total RNA was used as template for one step RT-PCR. The manufacturer's protocol was followed.

### ELISA

Cleaved Caspase-3 levels in same amounts of different cell lysates were detected with ELISA. The manufacturer's manual was followed.

### Stability of MFE standardized to α-mangostin in liver microsomes

0.5 mg/ml human liver microsomes (Invitrogen) and mouse liver microsomes (BD Biosciences) were incubated with the test substance, mangostin/MFE at a final concentration of 1 µM, in a solution containing 5 mM Isocitric acid, 5 mM MgCl_2_, 0.2 U/ml Isocitric Acid Dehydrogenase and 1 mM NADP+ in 100 mM Tris-HCl buffer (pH 7.4). The compounds were incubated with microsomes at 37°C for 0, 10, 20 and 30 mins in duplicate. Control tubes were incubated without NADP+. After incubation, samples were vortexed and kept on ice until ready for centrifugation at 17,000 g for 15 mins at 4°C. After centrifugation the supernatants were transferred into vials and analyzed using LC-MS method as described before.

### *In vivo* 22Rv1 tumor xenograft model

All animal experiments were performed in accordance with the guidelines approved by the Animal Care and Use Committee of the University of Illinois at Chicago. The protocol was approved by the animal care committee at the University of Illinois at Chicago (Protocol Number: ACC-11-019) to ensure steps were undertaken to ameliorate animal suffering. At the conclusion of the study all mice received general anesthesia by inhalation (i.e. isoflurane) followed by CO_2_ asphyxiation per the approved animal protocol for euthanasia. All animals were monitored on a daily basis in addition to animal care staff. Athymic (*nu/nu*) male nude mice (Harlan Laboratory, Madison, WI) seven-eight weeks old were housed under pathogen-free conditions with a 12 h light/12 h dark schedule and fed with an autoclaved AIN-76A diet *ad libitum* as described previously [9], [12]. 22Rv1 cells were used for determining the *in vivo* effects of MFE based on the fact that these cells form rapid and reproducible tumors in nude mice with tumor xenografts. Fourteen animals were randomly divided into two groups, with seven animals in each group. The animals in group 1 received vehicle (100 µL of cotton seed oil) by intraperitoneal (IP) administration and served as control. The animals in group 2 received mangosteen fruit extract (35 mg/kg) dissolved in cotton seed oil by IP two times weekly. Body weights were recorded throughout the study.

### Statistical analysis

All statistical analysis was performed by using VassarStats software. Data are expressed as mean with standard deviation for all groups. Statistical significance of differences in all measurements between control and treated groups was determined by one-way ANOVA followed by Tukey's HSD test for multiple comparisons. Student's paired t test was used for pair wise group comparisons, as needed. All statistical tests were two-sided, and P<0.05 was considered statistically significant.

## Results

### HPLC of mangosteen fruit extract

**Figure 1:**
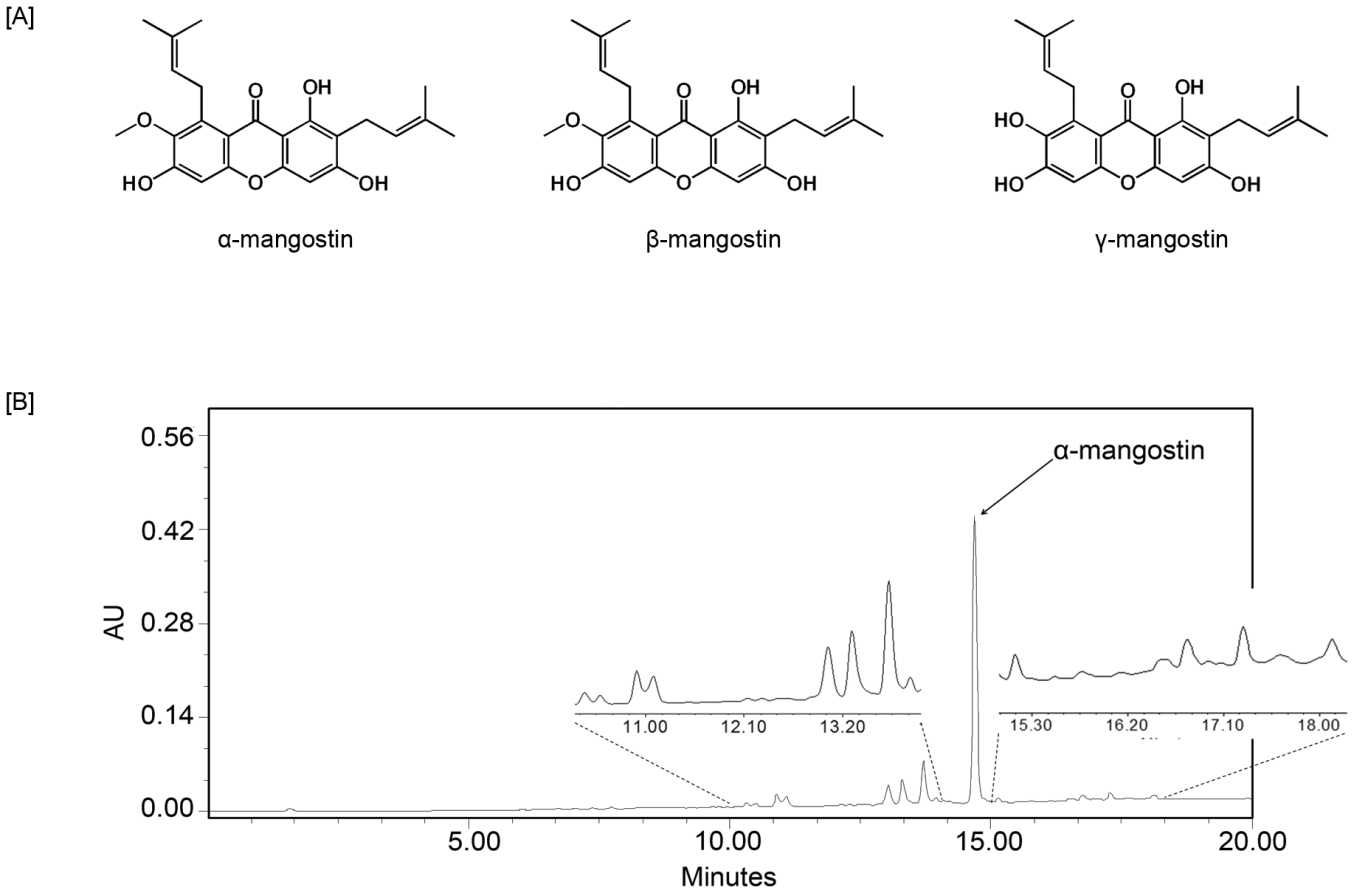
HPLC of Mangosteen fruit extract (MFE). [**A**], Polyphenolic xanthones isolated from the mangosteen fruit. [**B**], HPLC Chromatogram of mangosteen fruit extract. Sub-panel represents an expansion of the profile to reveal additional constituents in the extract.

Using HPLC we determined that MFE contained more than 35% α-Mangostin and was used throughout the study (Fig. 1A&B). Multiple peaks indicate that MFE also contains additional xanthones, such as β-Mangostin and γ-Mangostin (Fig. 1A&B).

### Mangosteen fruit extract reduced cell viability and cellular proliferation on prostate cancer cells

**Figure 2:**
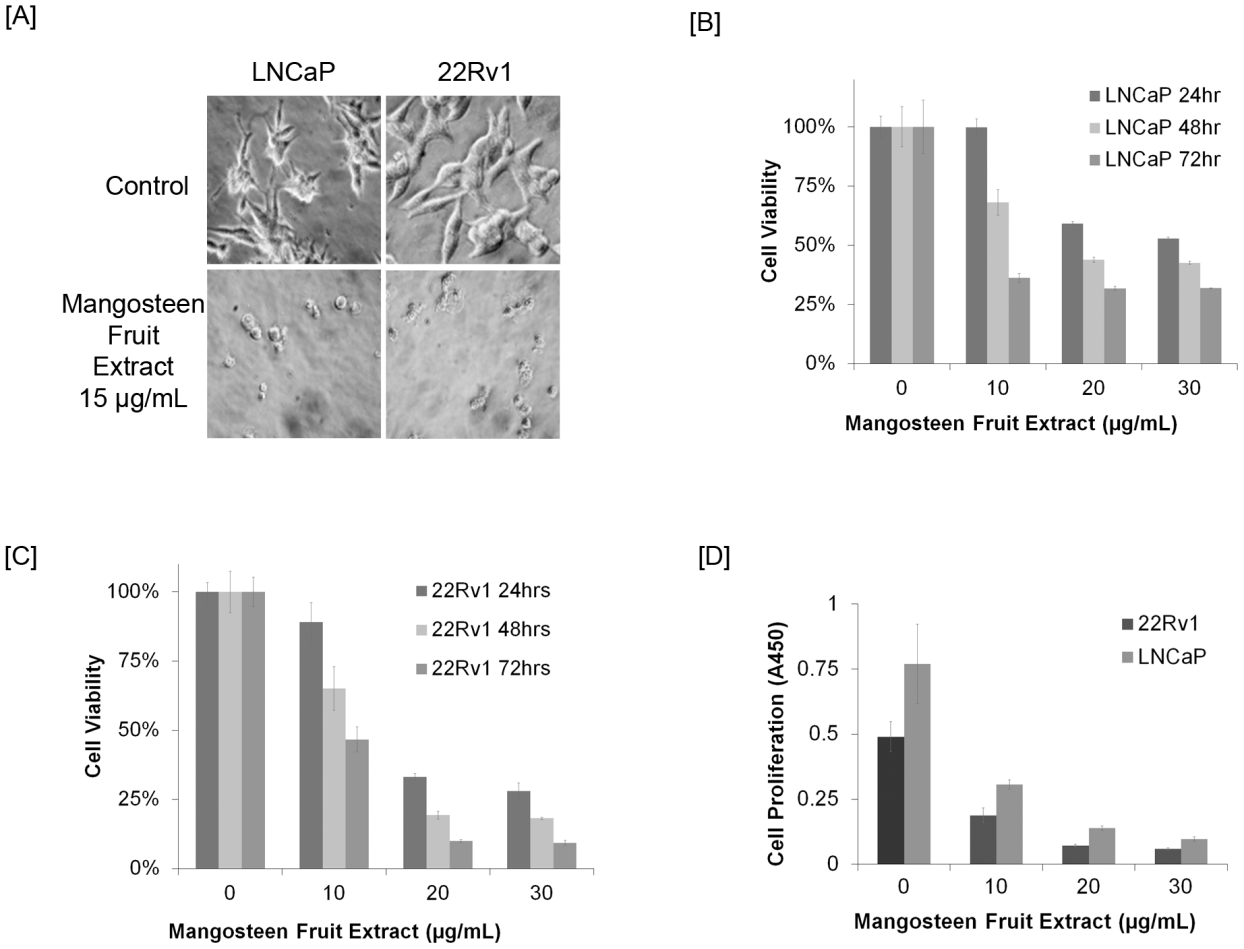
Mangosteen fruit extract (MFE) reduced viability and inhibited proliferation *in vitro* on two different prostate cancer cell lines, 22Rv1 and LNCaP. [**A**], Microscopic pictures of MFE-treated and untreated prostate cancer cells, MFE dose was indicated. [**B**], LNCaP cells were treated with increasing doses of MFE for 24, 48, or 72 hr, cell viability was determined by MTT assay. [**C**], 22Rv1 cells were treated with increasing doses of MFE for 24, 48, or 72 hr, cell viability was determined by MTT assay. [**D**], Both 22Rv1 and LNCaP cells were treated with increasing doses of MFE, cell proliferation was evaluated by BrdU assay. Absorbance values at 450 nm (A450) represent proliferating cell numbers. These experiments were performed in triplicate and are represented by the mean along with standard deviation.

We observed that MFE has obvious toxicity on both 22Rv1 and LNCaP cells under microscope (Fig. 2A). Using MTT assay, we observed that MFE decreased 22Rv1 and LNCaP cell viability in a dose- and time-dependent manner with a better effect on 22Rv1 cells (Fig. 2B&C). MFE also inhibited 22Rv1 and LNCaP cell proliferation in a dose-dependent manner (Fig. 2D).

### Mangosteen fruit extract induced apoptosis on prostate cancer cells

**Figure 3:**
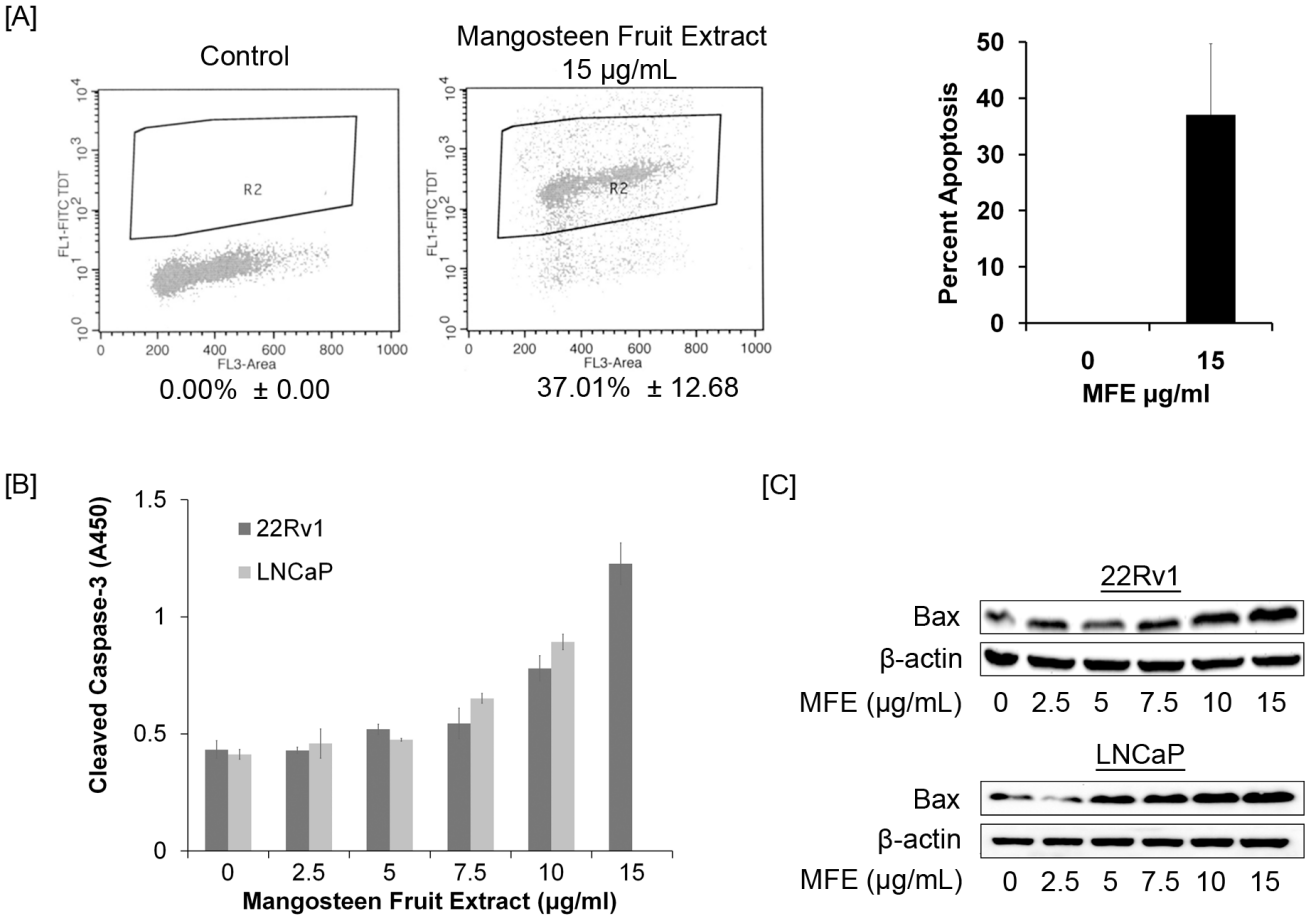
Mangosteen fruit extract (MFE) induced apoptosis and apoptosis-related gene expression in prostate cancer cells. [**A**], Percentage of apoptotic cells in MFE-treated and untreated cells were determined by flow cytometry. MFE dose was 15 µg/ml. [**B**], Cleaved Caspase-3 levels in increasing doses of MFE treated 22Rv1 and LNCaP cells. Cell lysates were prepared from MFE-treated cells and cleaved caspase-3 levels were evaluated from same amounts of cell lysates by ELISA, details see *Materials and Methods*. Relative expression levels were indicated as absorbance values at 450 nm (A450). [**C**], Bax expression in increasing doses of MFE-treated 22Rv1 and LNCaP. Cell lysates were prepared from MFE-treated cells, Bax expression was detected by western blot, and β-actin was used as a loading control. These results are representatives from three independent experiments.

At a dose of 15 µg/ml of MFE, the percentage of apoptotic 22Rv1 cells was average 37% determined by flow cytometry (Fig. 3A). Correspondingly, the level of apoptosis marker cleaved caspase-3 and the pro-apoptotic protein Bax expression in 22Rv1 and LNCaP cells increased with MFE doses (Fig. 3B&C). These results are consistent with our previous evaluation of pure α-mangsotin [9].

### Mangosteen fruit extract increased the expression of unfolded protein response proteins in prostate cancer cells

It has been well-established that ER stress activates UPR to reestablish cell homeostatsis. Thus, we examined the expression of important ER stress/UPR proteins in MFE-treated prostate cancer cells (22Rv1 and LNCaP). The expression of three important UPR proteins, pancreatic ER Kinase (PKR)-like ER kinase (PERK), inositol-requiring enzyme 1 (IRE1) and C/EBP homologous protein (CHOP), were gradually up-regulated with increasing doses of MFE on 22Rv1 and LNCaP cells (Fig. 3A).

### Mangosteen fruit extract modulated the ER stress-specific caspase-4

It has been reported that caspase-4 is localized to ER membrane and can function as an ER stress-specific caspase in human cells [14]. We detected cleaved caspase-4 in both 22Rv1 and LNCaP cells after treating cells with a range of doses of MFE for 24 hr. The results showed a dose-dependent increase of cleaved caspase-4 levels in MFE-treated cells (Fig. 3B).

### Mangosteen fruit extract modulated ER chaperones

We also investigated possible expression changes of ER chaperones in MFE-treated prostate cancer cells. BiP (immunoglobulin-binding protein), a critical ER chaperone involved in ER stress/UPR, was up-regulated with increasing MFE doses (Fig. 3C). Calnexin is an ER membrane protein for proper folding and quality control [15]. We observed a slight increase of calnexin expression from 0 to 10 µg/ml of MFE in both cell lines. Interestingly, calnexin expression decreased dramatically at 15 µg/ml of MFE in 22Rv1 cells (Fig. 3C). ER protein endoplasmic oxidoreductin-1 (Ero1-Lα) is an ER oxidase and up-regulated by CHOP to increase the levels of misfolded proteins [16]. MFE increased Ero1-Lα in a dose-dependent manner (Fig. 3C). MFE also increased another ER chaperone, PDI in a dose-dependent manner (Fig. 3C). These data demonstrated that MFE increased the expression of ER stress chaperones in prostate cancer cells.

### Mangosteen fruit extract induced spliced XBP-1 in prostate cancer cells

**Figure 4:**
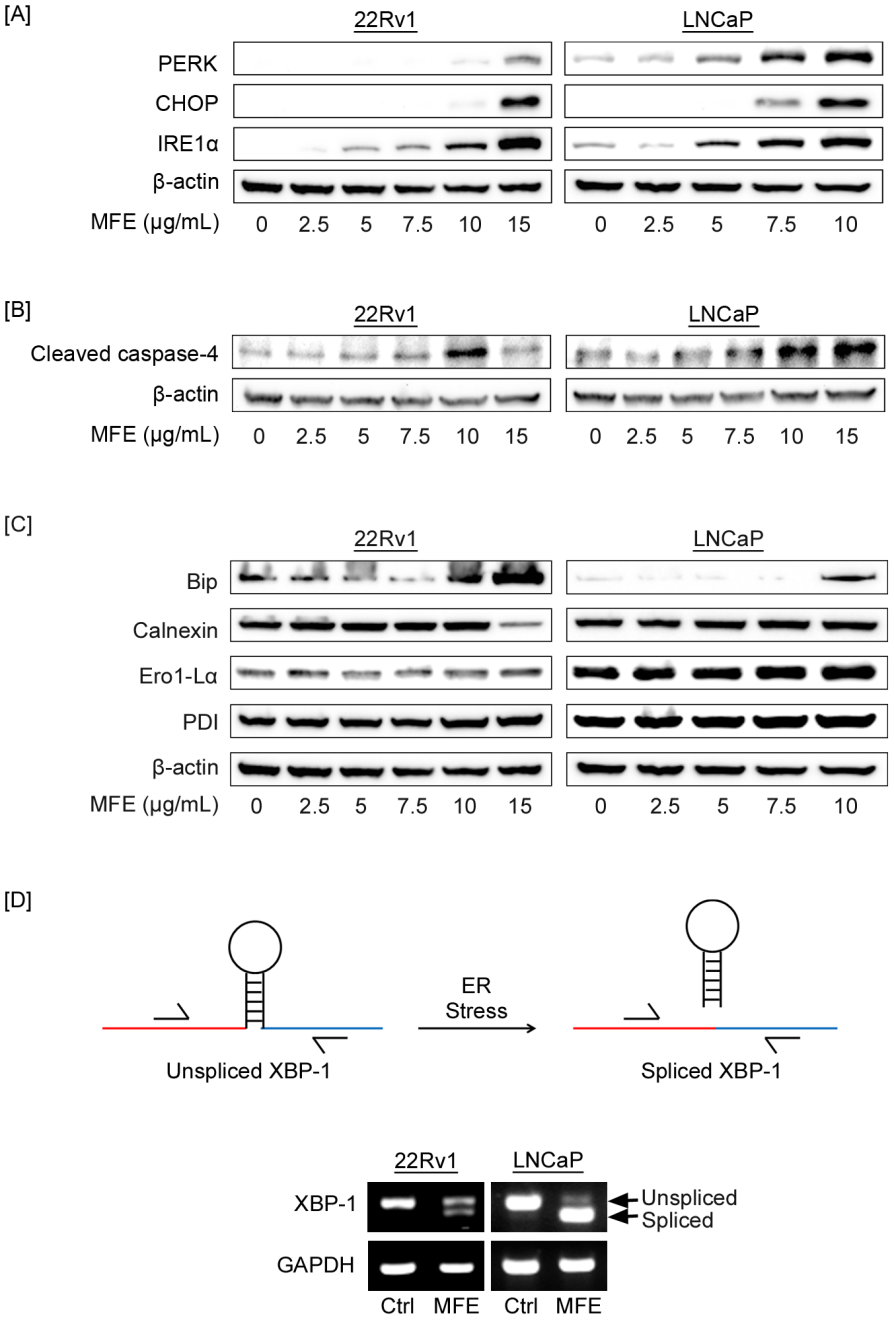
Mangosteen fruit extract (MFE) up-regulated endoplasmic reticulum (ER) stress proteins and chaperones in prostate cancer cells. 22Rv1 and LNCaP cells were treated with increasing doses of MFE or α-Mangostin for 24 hr. Cell lysates were prepared and subjected to western blot for detecting the expression of critical ER stress proteins. [**A**], Expression of ER stress proteins in increasing doses of MFE-treated 22Rv1 and LNCaP cells. [**B**], Cleaved caspase-4 levels in increasing doses of MFE-treated 22Rv1 and LNCaP cells. [**C**], Expression of multiple ER chaperones in increasing doses of MFE-treated 22Rv1 and LNCaP cells. Beta-actin was used as a loading control. These results are representatives from three independent experiments. [**D**], Upper panel, schematic diagram of XBP-1 splicing during ER stress. Lower panel, agarose gel pictures of RT-PCR products. 22Rv1 and LNCaP cells were treated with 15 µg/ml MFE for 24 hr. Total RNA was extracted and subjected to RT-PCR. XBP-1primers spanning the spliced sequence were used. RT-PCR products were run on a 2% agarose gel. GAPDH was used as a control.

During ER stress, X box binding protein-1 (XBP-1) mRNA undergoes splice to produce a transcription factor upregulating UPR genes. Thus we used RT-PCR to detect spliced XBP-1 in MFE-treated 22Rv1 and LNCaP cells. Spliced and unspliced XBP-1 cDNA were both observed in treated cells with only unspliced XBP-1 cDNA in control cells (Fig. 4D).

### Mangosteen fruit extract selectively induced ER stress in prostate cancer cells while sparing non-cancerous prostate epithelial cells

**Figure 5:**
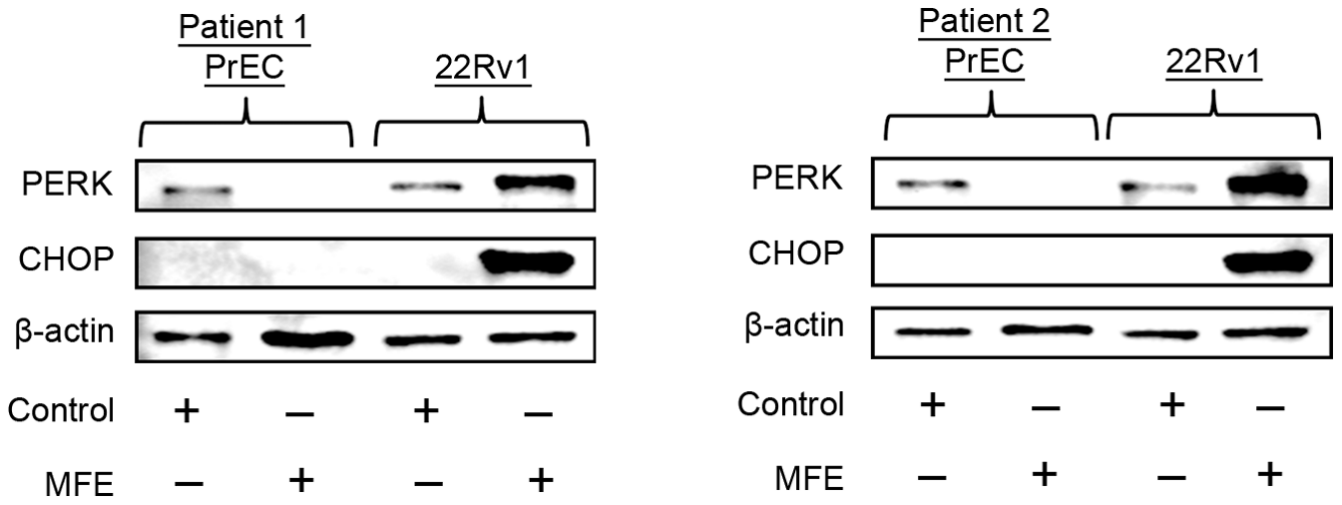
The effects of MFE on Prostate epithelial cells (PrECs) from prostate cancer patients. PrECs were isolated from patients undergoing radical prostatectomy. Samples were then used to prepare cells for treatment with study agents. PrECs from two different prostate cancer patients or 22Rv1 cells were treated with 15 µg/ml of MFE for 24 hr and then subjected to Western blots. Beta-actin was used as a loading control. Minus and plus symbols respectively represent absence and presence of indicated agents. These results are representatives from three independent experiments.

Next, our objective was to characterize the potential of MFE to promote ER stress in non-cancerous cells. Prostate epithelial cells (PrECs) were isolated from prostate biopsy on two prostate cancer patients undergoing radical prostatectomy. Cells were cultured to 60–70% confluence and then treated with MFE. Interestingly, 15 µg/ml of MFE significantly decreased PERK expression in PrECs from both patients, while same dose of MFE significantly increased PERK expression in 22Rv1. As a downstream protein regulated by PERK, CHOP expression did not increase in MFE-treated PrECs, while dramatically increased in MFE-treated 22Rv1 cells (Fig. 5). These data demonstrated that MFE selectively promotes ER stress/UPR in prostate cancer cells but not in non-cancerous prostate epithelial cells, suggesting a differential effect on modulating ER stress proteins in cancer cells versus non-cancerous cells.

### Stability of α-mangostin in mangosteen fruit extract in the presence of liver microsomes

**Figure 6:**
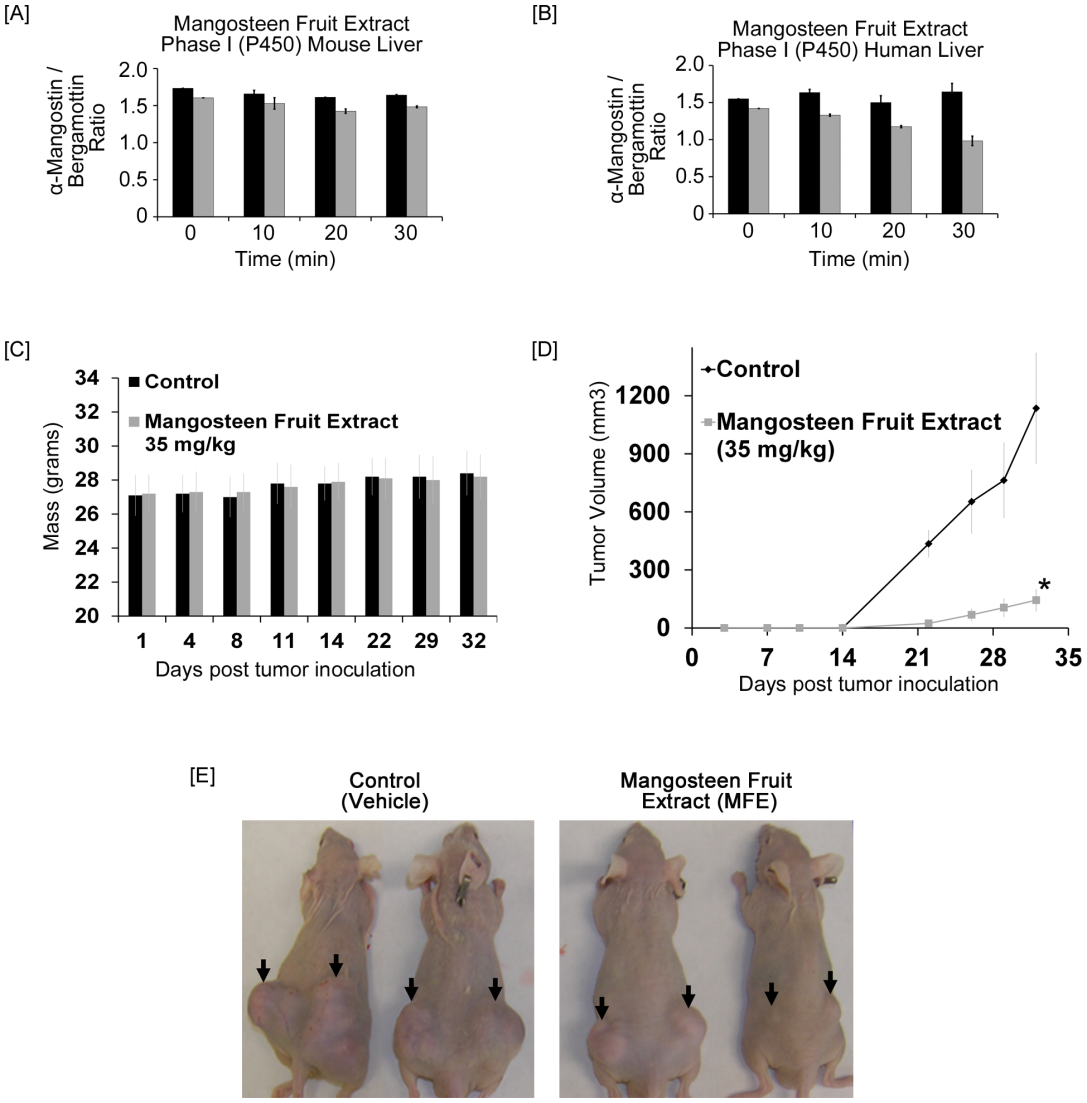
MFE was incubated with liver microsomes to mimic P450 Phase I metabolism in the mouse or human liver. Human and mouse liver microsomes were prepared as described in the materials and methods. Black bars represent the respective control for that individual time point. Gray bars represent the microsomal stability of α-Mangostin in Phase I enzyme system in both mouse and human liver microsomes. A ‘no preincubation’ was performed (data not shown) and did not have any impact on the interpretation of this data. [**A**], Mangosteen Fruit Extract (standardized to α-Mangostin) was incubated with mouse liver microsomes. [**B**], Mangosteen Fruit Extract (standardized to α-Mangostin) was incubated with human liver microsomes. [**C**], Twelve animals were subcutaneously injected in each flank of the mouse (i.e. two tumors per mice) with ∼1×10^6^ 22Rv1 cells to initiate tumor growth. Twenty-four hours after cell implantation, the animals in each cohort received by intraperitoneal administration vehicle or mangosteen fruit extract (35 mg/kg). Body weights of athymic nude mice treated with control vehicle or mangosteen fruit extract (35 mg/kg) intraperitoneally twice per week were measured two times weekly. [**D**], The average tumor volume of control and mangosteen fruit extract treated mice were plotted over days after tumor cell inoculation. Data points represent the mean of 12 tumors from six mice; bars represent standard deviation of the mean, *P<0.01. [**E**], Representatives of tumor bearing mice from control and MFE-treated group.

To characterize the extent of P450 metabolism, α-mangostin and mangosteen fruit extract was incubated in the presence of liver microsomes from mouse and human sources. For this particular experiment we evaluated liver microsomes as the administration of study agent. By 30 minutes, the stability of α-mangostin in MFE was found decrease slightly in mouse and human liver microsomes by 30.8% and 17%, respectively (Fig. 6A&B). These results suggest that Phase I metabolism has a minimal role in the metabolism of α-mangostin, which is the most abundant xanthone in MFE. These data suggested that Phase II metabolism in microsomes appears to be the primary route of MFE metabolism.

### Mangosteen fruit extract significantly inhibited the growth of human prostate cancer cell 22Rv1 in athymic nude mice without obvious toxicity

To examine MFE's anticancer activity *in vivo*, we constructed xenograft mice models with 22Rv1 cells and treated them with MFE or vehicle. Compared to control, 35 mg/kg of MFE significantly inhibited tumor growth (Fig. 6D&E). Meanwhile, no significant difference in body weights was observed between the MFE-treated group and the vehicle-treated group (Fig. 6C), suggesting MFE has low or no toxicity at an effective dose.

## Discussion

The mangosteen (*Garcinia mangostana*) fruit is reported to have a variety of pharmacological properties including increasing apoptotic indices in several difference cancer cell lines [17], [18]. A variety of polyphenols known as xanthones which consist of a tricylic aromatic ring system along with various isoprenyl, hydroxy, or methoxy groups have been isolated from the mangosteen. The most abundant xanthone is α-mangostin, an isoprenylated xanthone, which has received the most attention for its health promoting properties [9], [19]–[23]. When evaluating the fruit including the endocarp and exocarp along with the leaves, bark and roots more than 60 different xanthones have been isolated to date from the mangosteen. We have previously reported on the anti-cancer activity of α-mangostin in prostate cancer cells and an athymic nude mouse model [9]. In this previous study we observed α-mangostin to inhibit CyclinD1/CDK4 and suggest a possible mechanism for competitive inhibition in the binding site of CDK4. In this same study we also observed nude mice implanted with 22Rv1 cells to display a 65% smaller tumor volume following daily treatment with α-mangostin. In our experience, as well as other investigators, α-mangostin is well tolerated with some reports of doses as high as 200 mg/kg of body weight not being associated with any specific toxicity. Multiple reports along with our studies have observed α-mangostin to induce apoptosis, however, the events occurring prior to apoptosis are less understood. Furthermore, we have recently reported on the pharmacokinetic profile of orally administered α-mangostin in mice and have shown that the complete blood count (CBC) including white blood cell with differential was not altered [24]. For this reason we began to explore the endoplasmic reticulum as a potential target of a highly characterized mangosteen extract (>35% α-mangostin).

The endoplasmic reticulum is extremely sensitive to alterations in its surrounding environment that have a direct effect on its structure, integrity, and function [1], [2]. As cancer is promoted and progresses there is an increased demand on the requirements of the endoplasmic reticulum as evidenced by an increase in protein synthesis. This increased demand runs the risk of manufacturing proteins that are unfolded or misfolded. An important aspect of this increased demand on protein synthesis is the “unfolded protein response” that includes PERK, CHOP and several other ER stress proteins [25]. As homeostasis in the ER is “re-established”, proteins may be folded properly or undergo degradation that in turn will allow cells to survive. If homeostasis is not established by the unfolded protein response or is perturbed by an outside agent, these cells will undergo apoptosis. Therefore approaches to increase ER stress related protein response may be an attractive approach to alter the homeostasis of the ER in cancer cells in order to promote apoptosis. The evidence by other investigators and ourselves suggests that the use of small molecules that modulate ER stress proteins may also retard tumor development, growth, invasion, thereby providing a novel therapeutic strategy [26], [27].

ER stress not only favors the survival of cancer cells, but under specific situations can promote apoptosis. Natural agents, such as curcumin from the turmeric (*Curcuma longa*) have been reported to induce pro-apoptotic ER stress in human leukemia HL-60 cells by upregulating the expression of phosphorylated PERK, CHOP, Bip and cleaved caspase-4 [28]. Consistent with this, in our study following treatment with MFE both 22Rv1 and LNCaP cells undergo ER stress as evidenced by an increase in ER stress proteins including PERK, CHOP, IRE1α, Bip and cleaved caspase-4. CHOP is a well-established downstream ER stress/UPR protein and plays an important role in ER stress-induced apoptosis [29]. The mechanisms that CHOP contributes to apoptosis include inhibiting anti-apoptotic Bcl-2 and up-regulating GADD34 and Ero1α. Up-regulation of GADD34 and Ero1α will aggravate ER stress by translation recovery and increasing misfolded protein levels. In 22Rv1 cells, the most dramatic increase of CHOP occurred at the dose of 15 µg/ml MFE. Coincidentally, calnexin, an ER membrane protein ensuring proper folding and quality control, dropped remarkably at 15 µg/ml MFE treatment. This suggests that 15 µg/ml MFE caused a possible breakdown of the quality control system of ER. Also, PERK, IRE1 and Bip all underwent a striking increase at 15 µg/ml MFE treatment. Taken together, these data suggest that 15 µg/ml of MFE might be an ideal dose to push ER stress from pro-survival to pro-apoptosis in prostate cancer cells.

The next step was to understand if MFE will result in ER stress in non-cancerous cells or whether there appear to be a selective effect towards cancer cells. To understand this further, prostate epithelial cells were isolated from two human subjects undergoing radical prostatectomy. The effect of MFE was examined on benign primary prostate epithelial cells (PrECs) and 22Rv1 cells. In contrast to immortalized prostate cancer cell lines treated with MFE, which result in an increase in PERK activity, PrECs showed a decrease of PERK. This biphasic response dependent on the cell type further illustrates the complexity of ER stress in prostate cancer carcinogenesis. Taken together, the evidence suggests that MFE selectively target prostate cancer cells while sparing normal cells. A further and more detailed understanding of this differential effect will be required.

As we have previously described α-mangostin when administered orally resulted in a 65% smaller tumor in 22Rv1 cells [9]. In 22Rv1 implanted mice treated with MFE we observed an 88% smaller tumor volume when compared to control mice. The dose used in this experiment was 35 mg/kg of mangosteen fruit extract which approximates to 12 mg/kg of α-mangostin. To understand if α-mangostin is the primary constituent of mangosteen fruit extract 22Rv1 cells were treated with 35 mg/kg and 70 mg/kg of α-mangostin. The treatment regimen with only α-mangostin represents a 300 to 600% increase in α-mangostin exposure. Even at 70 mg/kg of α-mangostin, which resulted in a 69% smaller tumor, the pure phytochemical was not as effective as MFE containing 12 mg/kg of α-mangostin (unpublished data). In retrospect, this is not that surprising as a mixture of phytochemicals are often reported to have a synergistic effect that is superior to individual phytochemicals. As evidenced by our chromatogram there are a variety of xanthones present in our extract, possibly greater than 20 additional xanthones, which could be responsible for the superior anti-cancer properties of a mangosteen extract compared to an individual xanthone.

The accumulation of germline and/or somatic mutations in “normal” cells results in an inability to overcome cell cycle and apoptotic checkpoints thereby resulting in the initiation of cancer. As the proliferation of cells increases an increased demand on the protein machinery of a cell must adapt through various mechanisms including endoplasmic reticulum stress. In our studies we have observed mangosteen fruit extract standardized to α-mangostin to selectively induce ER stress in prostate cancer cells which correlates with an increase in apoptotic indices. Interestingly, in normal prostate epithelial cells procured from patients at high risk of developing prostate cancer MFE is observed to decrease ER stress. Our mangosteen fruit extract contains >35% α-mangostin, future experiments are investigating whether α-mangostin is the primary xanthone responsible for these properties.

## Supporting Information

Figure S1 **Colony formation was performed using 22Rv1.** For colony formation, cells were plated at ∼1,000 cells per well and incubated for 48 h. After 48 h, media was replaced with fresh media containing mangosteen fruit extracts along with vehicle controls. This was repeated every 3–4 days until completion of the experiment.
(TIF)
