# Supplementary figures and images for: Selective Modulation of Endoplasmic Reticulum Stress Markers in Prostate Cancer Cells by a Standardized Mangosteen Fruit Extract
Source: PLoS One. 2013 Dec 18;8(12):e81572. doi: 10.1371/journal.pone.0081572 (PMC3867317; doi:10.1371/journal.pone.0081572)

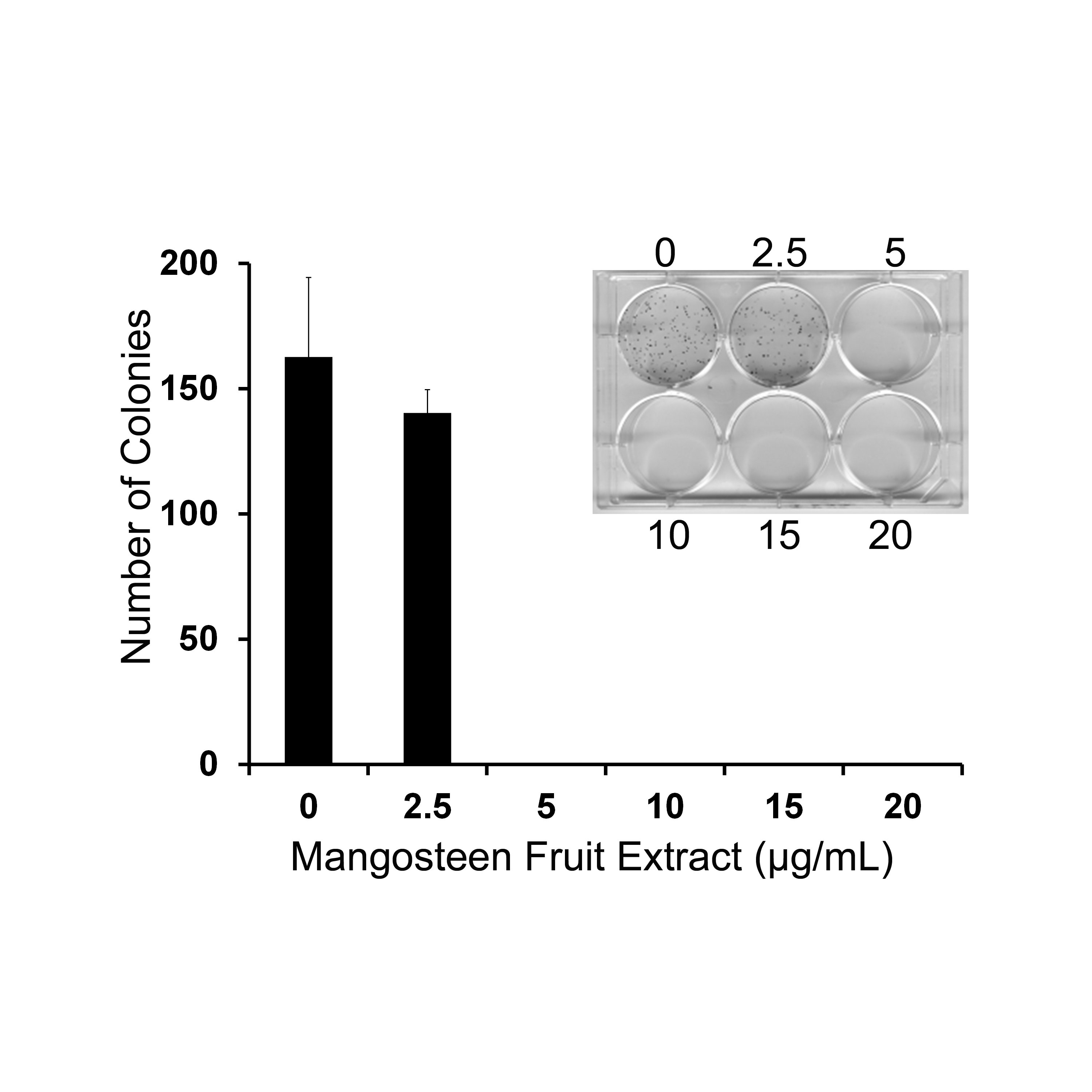

Supplement: Figure S1 — Colony formation was performed using 22Rv1. For colony formation, cells were plated at ∼1,000 cells per well and incubated for 48 h. After 48 h, media was replaced with fresh media containing mangosteen fruit extracts along with vehicle controls. This was repeated every 3–4 days until completion of the experiment. (TIF) [file pone.0081572.s001.tif]
